# The Effect of the Fuel Location and Ventilation Factor on the Fire Dynamics of Informal Settlement Dwellings

**DOI:** 10.1007/s10694-023-01517-1

**Published:** 2023-12-21

**Authors:** M. Beshir, Y. Wang, A. Cicione, R. Hadden, M. Krajcovic, D. Rush

**Affiliations:** 1 Center for Fire Safety Engineering, School of Engineering, University of Edinburgh, Edinburgh, EH9 3JL UK; 2 Department of Civil and Environmental Engineering, Carleton University, 1125 Colonel By Drive, Ottawa, ON K1S 5B6 Canada; 3 State Key Laboratory of Fire Science, University of Science and Technology of China, Hefei, 230026 People’s Republic of China; 4 Department of Civil Engineering, Stellenbosch University, Cape Town, South Africa

**Keywords:** Compartment fire, Informal settlement dwellings, Under-ventilated, External heat flux, Fuel location, Ventilation factor

## Abstract

Three full-scale experimental compartment fires are compared to investigate the effect of the fuel location and the ventilation factor on under ventilated thermally thin bounded ISO-9705 compartments. Wood cribs were used as the fuel load and the crib placement was varied between two locations (back and middle) to study the effect of the fuel location. Furthermore, the ventilation conditions were changed from a door and window (i.e., ventilation factor of 2.58 m^5/2^) to only a door (i.e., ventilation factor of 2.26 m^5/2^) for the scenario where the cribs were placed at the back of the compartment. The novelty of this work lies in its examination of the time to flashover, gas layer temperature, heat release rate, and external radiative heat fluxes, specifically considering the impact of fuel location and ventilation factor. It was observed that placing the fuel package in the middle of the compartment led to a longer growth phase, hotter gas layer temperature, a higher Heat Release Rate (HRR) needed for flashover ($\dot{q}_{fo}$), and higher external radiative heat fluxes through openings. It was also found that, decreasing the ventilation factor decreased the heat losses and therefore the $\dot{q}_{fo}$. Decreased ventilation also affected the height of the neutral plane, as one would expect, and the shape of the external plume, but did not have significant effect on the temperature within the compartment, the walls of the compartment, and the external radiative heat flux.

## Introduction

The phenomenon of urbanization has emerged as a significant global challenge in the twenty-first century, encompassing crucial aspects such as housing, infrastructure development, safety measures, and the interface between urban areas and wildland environments [1]. In the past few decades, most of the urban growth occurred within the Global South (GS). It is estimated that each year the urban areas in the GS, more specifically the Low/Middle Income Countries (LMIC), grow by 70 million people [2]. With these increases, authorities struggle to keep up with the housing demand. This leads to Informal Settlements (ISs) being established by the urban vulnerable, with now more than one billion people currently living in ISs across the globe. This number is ever increasing, with ISs residents increasing by around 213 million people between1990 and 2015. This puts the ISs population at around 25% of the total current global urban population. For more specific country-based statistics, in South Africa it is estimated that up to 33% of the country’s population now live in IS. In Cape Town, the number of IS dwellings (ISDs) increased from 28, 000 in 1993 to 220, 000 in 2011. In Brazil statistical studies showed that there are approximately 11 million people living in IS which is about 6% of the total country’s population [3]. Inhabitants of ISs use readily available materials to build their homes, with the final design not complying with the formal building codes, thus and leave the residents vulnerable to hazards such as fires.

In the last few years there were many destructive fires around the globe, specifically in the GS (e.g., Imizamo Yethu, Western Cape, South Africa (2017) [4], Dhaka, Bangladesh (2019) [5] and Bahay Toro, Philippines (2011) [6]); these fires led to hundreds of deaths and thousands of homeless people.

Informal settlement dwellings (ISDs) are different in each country, and they are all also different from the formal compartments and dwellings in many aspects. For example, in South Africa, dwellings’ walls and roofs are usually made from thermally thin (e.g., steel sheets) or combustible (e.g., timber) materials; due to the poor construction, gaps/leakages usually exist between the walls; these gaps are often then filled with combustible materials (e.g., cotton cloth) and to provide wall insulation combustible lining materials are often used (e.g., cardboard).

Giving these different factors, the compartment fire dynamics of ISDs is also different than the well-studied “normal” compartments of the Global North. Since 2017, there have been many scientific studies (both numerical and experimental) to fill the gap of knowledge for these unique compartments and to understand the different fire spread mechanisms within ISs. Studies were done on different scales, from bench scale material tests (e.g., [7]), small scale experimental and numerical compartment fires to study the heat transfer and Heat Release Rate (HRR) onset of flashover (e.g. Ref. [8]), large outdoor full-scale compartment fire experiments to understand the difference in fire dynamics and spread between timber and steel cladding ISDs (e.g., Refs. [9–12]), and indoor lab-based large scale compartment fire experiments with steel clads to understand the effect of different boundaries on the fire dynamics and heat fluxes to the surroundings from the dwelling of origin (e.g., Ref. [13]). Numerically, these works were then modelled using the Large Eddy Simulation (LES) software Fire Dynamics Simulator (FDS) [14] where the utility of FDS in capturing the fire dynamics within the ISDs was discussed (e.g., Ref. [15]), and the effect of wind on the onset for flashover in ISDs was investigated numerically using FDS and small scale compartment fires (e.g. Ref. [16]). These studies fed into fire risk mapping using remote sensing and GIS techniques (e.g., Ref. [17]) and for fire spread modelling (e.g., Ref. [18]), to help determine the critical separation distance between dwellings (e.g., Refs. [12] and [19]). Whilst Beshir et al. [20, 21] studied numerically the effect of adding horizontal openings to the ceiling of the full-scale ISDs to see its impact on reducing eternal fluxes and thus decreasing the critical separation distance.

This study explicitly looks at two aspects, namely, the effect of the ventilation factor and location of the fuel package within the ISDs on the fire dynamics and the external radiation from the dwelling of origin. The primary motivation for investigating the location of fuel is to establish a foundation for future experiments and comparisons. By quantifying the effects of fuel location within the compartment, we enable researchers to easily compare results across different experiments where fuel placement may vary. This standardization allows for more meaningful comparisons and a better understanding of the role of fuel location in fire behaviour within informal settlements. Furthermore, the investigation into the interaction between ventilation factors and internal fire dynamics in informal settlements dwellings is motivated by several factors. Firstly, informal settlements are often characterized by thermally thin bounded compartments, which differ from more conventional structures. Therefore, it was aimed to determine if the influence of ventilation factors in such settings differs significantly from standard compartments. Secondly, considering the presence of wall leakages in informal settlements, the study sought to assess whether ventilation factors might yield different effects in these dwellings compared to more typical structures. This exploration is vital in comprehending the unique fire dynamics and risks associated with informal settlements, as well as potentially informing safety measures and interventions tailored to these specific environments.

The study will be based on two new large scale (using the same internal dimensions of the compartments used in ISO-9705 [22]) lab-based steel-clad compartment fire experiments, with wood cribs as fuel load; and comparing these experiments to the Base Line (BL) case presented by Wang et al. [13] (also discussed below). The aim of the study is to understand the significance or not of fuel location and ventilation on the expected fire spread parameters, such as external heat fluxes, when conducting large scale compartment fire experiments. The study is mainly experimental but numerical investigation using the Computational Fluid Dynamics (CFD) model namely FDS is used to further understand some aspects while comparing the fire dynamics of different compartments. The numerical investigation is based on a validated FDS model for these types of compartments, with the modelling methodology and inputs were presented in details by Beshir et al. [15]. Utilizing Computational Fluid Dynamics (CFD) models, rather than zone models, offers several advantages when studying fire dynamics in informal settlement dwellings (ISDs). CFD models provide greater accuracy, handle geometric complexity, analyze ventilation effects, and allow for validation and sensitivity analyses, making them invaluable for understanding complex fire behavior in ISDs. Additionally, the validation of CFD models enhances their reliability and application. These models are versatile tools that have been used to investigate the impact of atmospheric wind on fire severity in ISDs, providing essential insights for fire safety design and mitigation strategies in these unique environments.

## Experimental Setup

### Dwelling Design

Two dwellings with identical internal dimensions to ISO-9705 room [22] (3.6 m × 2.4 m × 2.4 m), were constructed. These two dwellings are part of a series of 13 experiments [13], investigating the fire dynamics and fire spread characteristics within IS. The walls and ceiling (boundaries) were made out of galvanized steel sheets that had a thickness of 0.51 mm and were attached together using timber frames, which were 0.038 × 0.089 m in cross-section. The idea behind this design was to mimic dwellings found in ISs of the Western Cape, South Africa.

**Figure 1:**
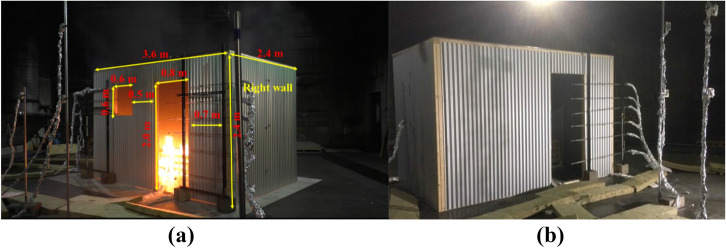
(a) BF (window + door openings), left, and (b) NW (door only openings), right

As presented in Figure 1a, in the first dwelling configuration, two openings were placed on the front wall, including a door with internal dimensions of 2.0 m (height) × 0.8 m (width) and a window of internal dimensions of 0.6 m (height) × 0.6 m (width). The door and window were 0.7 m and 2.0 m away from the right front corner, respectively. This dwelling was labelled as BF (i.e., Back Fuel which is identical to the Base Line—BL- compartment presented in [13] but with fuel placed in the back, whereas the BL scenario had the fuel package in the middle of the dwelling). The second dwelling was almost identical to the BF scenario, with the only difference being that this scenario only had one opening, the door. This dwelling was labelled as NW (i.e., No Window), as presented in Figure 1b. Therefore, the ventilation factors ($$V_{f} )$$ for the two BF and NW dwellings are 2.58 m^5/2^ and 2.26 m^5/2^, respectively. It should be noted that both openings were kept open during the experiments and were not covered with a door or glass.

**Figure 2:**
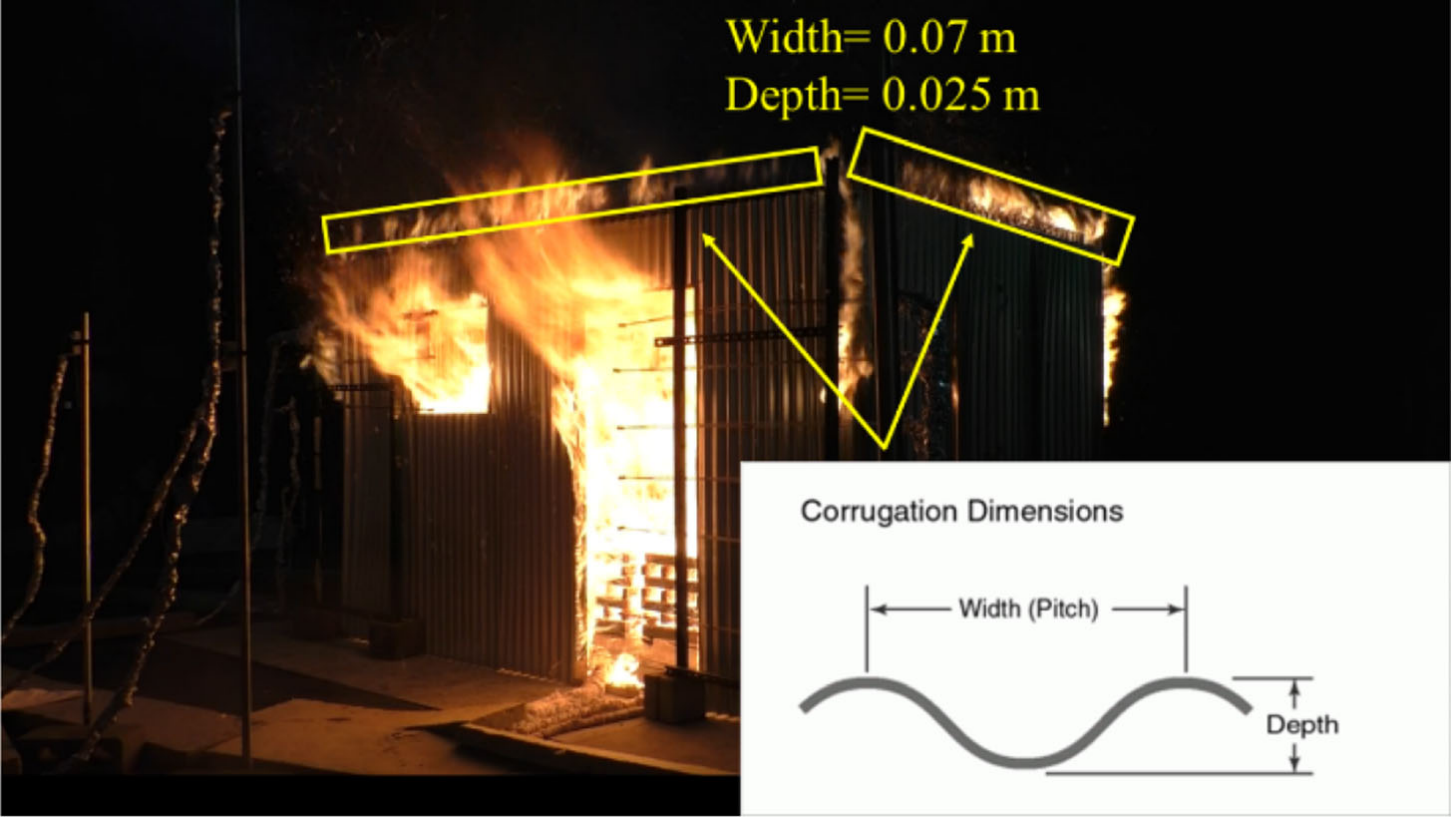
Corrugated steel leaking external flaming and a schematic of the corrugation’s dimensions with width and depth of each corrugation

The poor construction of the ISDs leads to the presence of gaps between the walls and the walls with the roof. These gaps leak out hot gases during compartment fires and is considered a unique parameter of these compartments. In Figure 2, the corrugation dimensions of the steel sheets used are presented. Figure 2, further illustrates the leaked hot unburned gases mixing with the air (oxygen) outside of the compartment, reaching the stoichiometric ratio mixture and hence the observed external flames close to the steel walls corrugations. These leakage gaps were left un-filled in all experiments presented in this paper. The impact of filling these gas with ceramic fibre blanket on the fire dynamics was investigated experimentally in [13] and numerically in [15].

The fuel load was designed to be around the average fuel load found in ISs in Western Cape, South Africa based on surveys conducted in 2017 [23]. The fuel load was made out of a wood crib (224 kg) placed at the centre next to the back wall of the compartment. The wood crib consisted of 7 layers of 10 sticks with a dimension of 0.038 × 0.064 × 2.44 m^3^ and density of 540 kg/m^3^. The sticks were placed with the short edge of the cross-section being horizontal. Therefore, assuming a heat of combustion of wood to be around 17.5 MJ/kg [24], the fuel load in these experiments was approximately 437.5 MJ/m^2^.

The decision to use wood cribs as the fuel load was based on several main reasons as Comparability: Wood cribs have been widely used in previous compartment fire studies reported in the literature, by using wood cribs, we can easily compare our results with those from existing studies, enabling a more comprehensive understanding of the fire dynamics in similar scenarios; Realism: Furniture, which is predominantly made of wood, represents one of the main fuel loads present in dwellings. By using wood cribs, we aim to simulate and study the fire behaviour associated with this common fuel type encountered in real-life situations; Simplified Modelling: Wood cribs offer a straightforward representation of fuel, with readily available thermal and physical properties in literature (e.g., Refs. [9–12]). This facilitates the modelling process, particularly when utilizing computational fluid dynamics (CFD) simulations. The availability of validated wood crib models allows for better model validation and a more accurate understanding of compartment fire dynamics using computer models; Empirical Correlations: Many existing empirical correlations for compartment fires are based on studies that utilized wood cribs as the fuel load [24]. By using wood cribs in our experiments, we can assess the validity of these correlations within the context of urban informal settlement conditions and potentially update them if necessary, using similar methodologies; and Soot Considerations: Wood cribs tend to produce cleaner fires with less soot compared to high carbon-based fuels like polypropylene. This allows for easier experimental observations and reduces the complexity associated with measuring and modelling soot, which remains a significant challenge in compartment fire research.

The ignition source was mop head strips soaked in Gasoline-87, which were then placed in a small plastic bags (to prevent the gasoline from evaporating. Six bags used in total; one in each of the four crib corners, one placed in the middle front (closest to door side) of the crib and one placed in the middle back (closest to back wall) of the crib.

### Measurements

**Figure 3:**
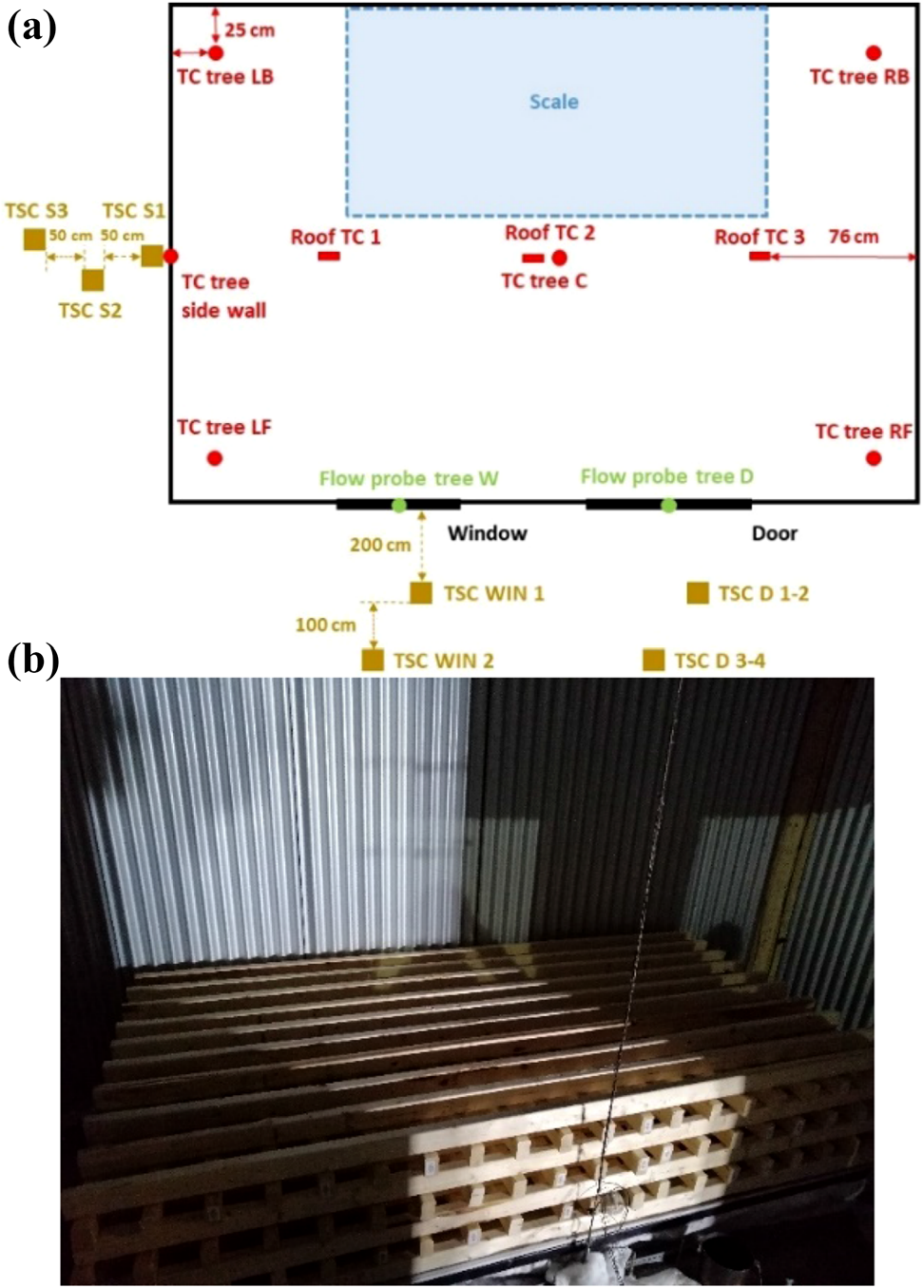
The plan view of the crib and measurements’ locations, not to scale, (a) and wood crib internal design (b)

**Figure 4:**
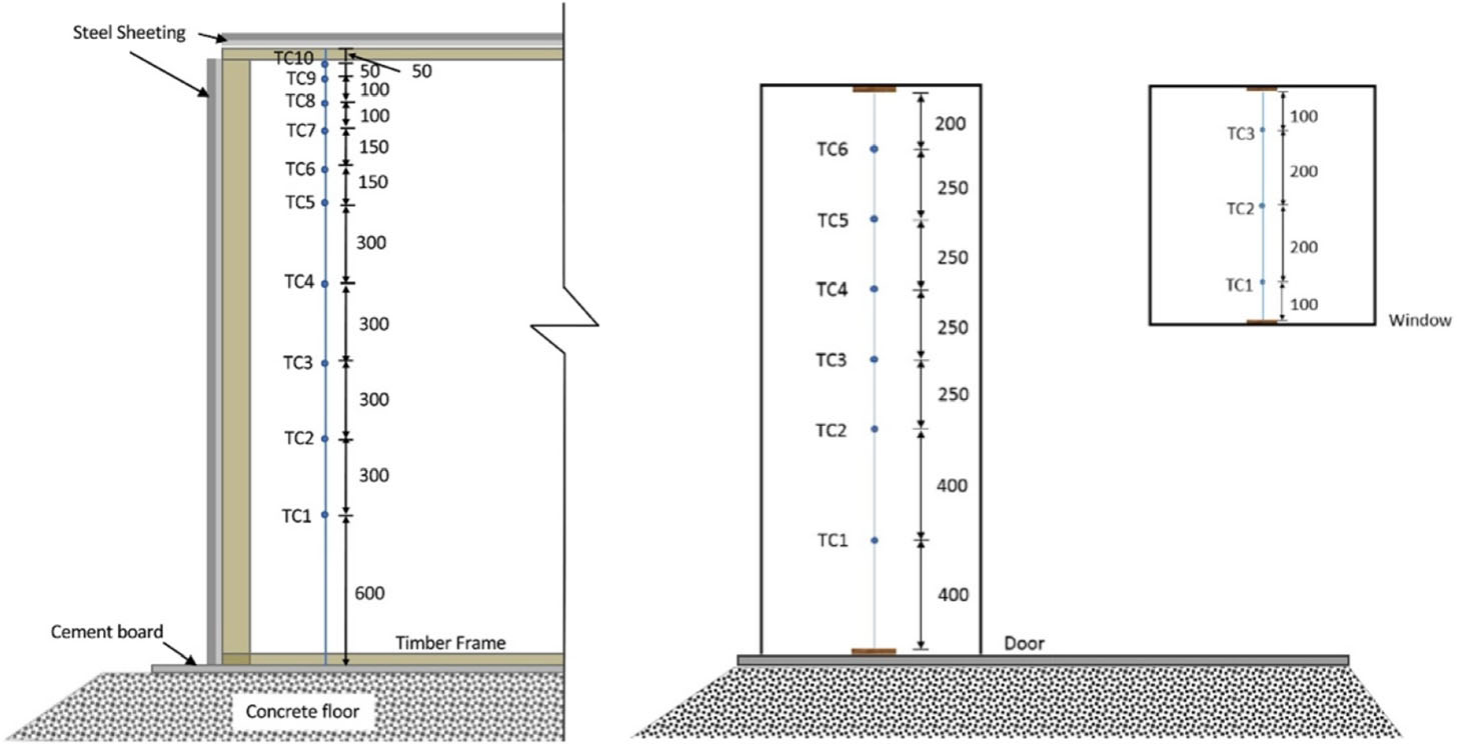
The design of the TC trees (left image) and the flow probes (right image) replicated from [13]

As presented in Figure 3, the wood crib was placed on top of a 2.0 m × 2.0 m scale at the back of the compartment. The scale has a 0.1 kg precision and was used to measure the mass loss of the wood crib. Five thermocouple (TC) trees were hung from the ceiling to the floor, one was placed in each of the compartment’s corners, and one was placed along the centreline of the long axis of the ceiling. Each TC tree consisted of 10 Inconel sheathed Type-K thermocouples with a tip diameter of 1.0 mm. The flow velocity along the vertical centreline of the door and window were measured using six and three bi-directional flow probes with an associated thermocouple, respectively. The roof external temperature was measured using three TCs at three locations (rear, middle and front). The design of the thermocouple trees and flow probes are presented in Figure 4.

To capture the irradiation (incident radiant heat flux) from the openings’ external plumes, Thin Skin Calorimeters (TSCs) [25] were placed outside the compartment. Four TSCs were placed in front the centre of the door; two at 1.6 m and two at 2.5 m at distances of 2.0 m and 3.0 m. Two TSCs were placed in front the centre of the window at a height of 1.6 m and same distances 2.0 m and 3.0 m. Three TSCs were placed next to the left wall at distances 0.0, 0.05 m and 1.0 m to capture radiative heat fluxes from the wall. The experiments were conducted under a pre-calibrated large fire calorimeter hood with a height of 7.62 m off the floor and a hood inlet diameter of 7.62 m, located in the burn hall of Underwriter Laboratories, Chicago, USA. The hood’s fan extraction rate was controlled to vary between 12 and 16 m^3^/s during both experiments and the convective and total HRR were recorded every second (Note that all the presented HRR values in this paper is based on the total HRR recorded via the hood). The baseline (BL) case [13] used for comparison, was of the exact same design as BF except for the fuel load, which, for the BL case, was placed in the middle of the compartment and consisted of two wood cribs with a distance of 0.18 m between the two cribs with more details presented in [13].

## Results and Discussion

The results are presented and discussed in two sub-sections: (a) the effect of the fuel location, and (b) the effect of the ventilation factor, both on the internal fire dynamics (total HRR, gas layer temperature and flow through openings flow) and the external radiation (the radiative heat flux from the openings and the walls).

### Fuel Location

#### Experimental

In this section, the effect of the fuel location in two identical under-ventilated compartment fire experiments is investigated. The two scenarios in this study are the baseline (BL) scenario as presented by Wang et al. [13] and the BF scenario as described above.

**Figure 5:**
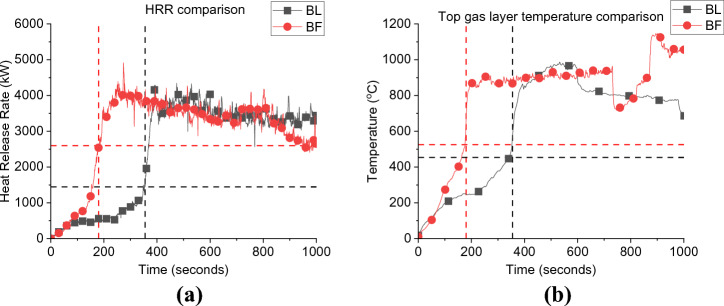
Comparison between BF and the BL case for; (a) Heat Release Rate; and (b) Top gas layer temperature, where the dashed lines present the time and HRR/Temperature at flashover for each case

Considering Figure 5a, it is observed that by placing the fuel package at the back of the compartment (i.e., BF) leads to a faster onset of flashover compared to the scenario where the fuel package was placed in the centre of the compartment (i.e., BL). Flashover occurred at approximately 206 and 355 s for BF and BL cases, respectively. Flashover, in this study, is defined as the sudden propagation of flames out of the compartment. As noted in the earlier work by Beshir et al. [15] this led to a significant observation. The conventional hot gas layer temperature criterion, typically falling within the range of 500 to 600°C, may not be directly applicable to the unique compartments found within informal settlements. This finding led the study to reorient its focus towards a different indicator—the presence of external flames. By shifting the attention to the emergence of external flames, the study aimed to identify the critical transition from the growth phase to the under-ventilated phase within these specific compartments. This transition holds substantial importance in comprehending the intricacies of fire behaviour and dynamics in informal settlement dwellings.

The external plumes were tracked using recorded videos for each experiment and the exact time for flashover was recorded based on this criterion. Therefore, the HRRs required for the onset of flashover ($\dot{q}_{fo}$) for the BF and BL scenarios are estimated to be around 2597 and 1445 kW, respectively. Note that for the BL case, eight, rather than six, ignition sources were used, and that the fuel package was separated into two distinct cribs compared to the BF single crib. This shows that placing the wood crib at the back of the compartment increases the severity of the fire.

The faster flashover for the BF scenario can be explained that, given the same ventilation factor, when the fuel is placed near the wall before flashover (e.g., at fuel-controlled period), the area available for the air to entrain through the wood crib was reduced. This restriction of air entrainment to the wood crib led to an increase in the flame length of the already ignited wood sticks due to the excess volatilized fuel. Therefore, the fire became under ventilated earlier in time than the centred crib case (BL) [26]. These results confirms what was previously found in thermally thick bounded compartment fire experiments (i.e., [26]) and therefore, there is no significant differences between thermally thick and thermally thin bounded compartments in this sense. In addition, the peak HRR value was also found to be almost similar for both cases (e.g., around 3.7 MW). Due to technical issues, all the corner thermocouple trees in BF failed after the first few seconds during the experiment and only the centre TC tree survived for the full duration. For the BL case, there was no middle TC tree as the wood crib was placed in the middle of the compartment, however, there was a TC tree in the middle of the compartment at front (i.e., near the door/window wall). As demonstrated in Figure 5b, the peak gas layer temperature for both cases was around 890 °C and there were no observed variations except for the growth phase which is expected to be less steep for the front thermocouple tree for the BL case as it placed closer to the openings which leads to a continuous cooling down of the gas layer at this location.

**Figure 6:**
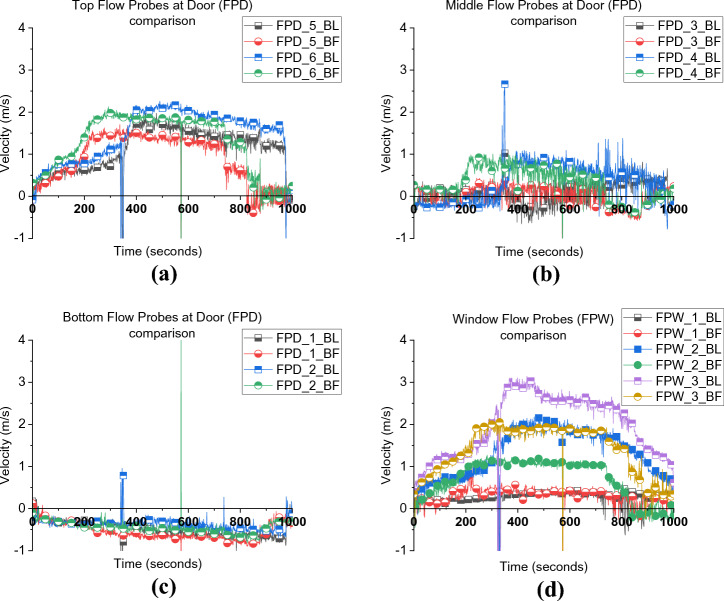
Flow probes (a) door top 5, 6, (b) middle flow probes 3, 4, (c) bottom flow probes 1, 2, and (d) window flow probes 1, 2, 3 for both case_1 and BL

**Figure 7:**
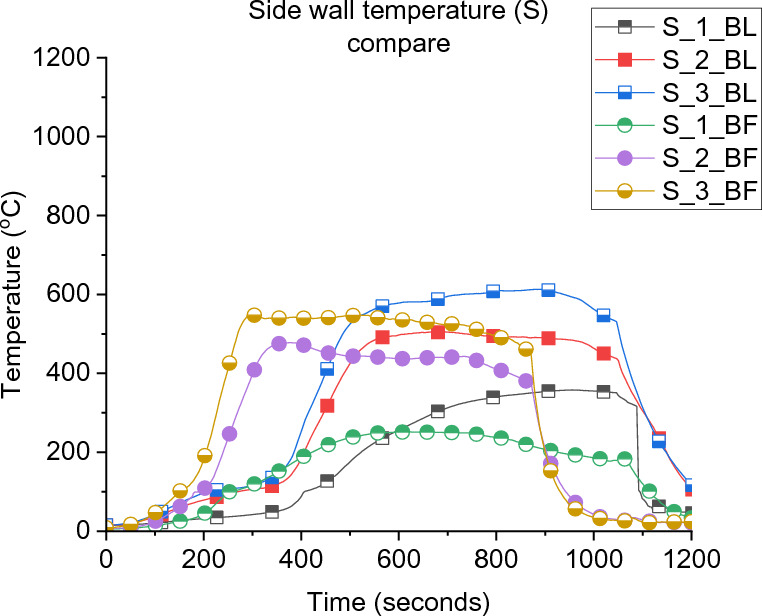
Side wall temperature distribution

Figure 6 presents the velocity of the inlet and outlet gases through the door and window. In both cases, it was found that the top 4 door flow probes experienced outlet flow while the bottom two were inlet flow. This allows the estimation of the neutral plane, which for both cases are approximately at the same location of around 0.8 to 1.05 m from the floor (≈0.9 m). However, the velocities of the top two flow probes of the door and the window flow probes in the BL case were slightly higher than those in BF. This phenomenon can be explained if one considers the wall temperature distribution for both cases, as depicted in Figure 7. It is clear that BF experienced slightly lower wall temperatures, which reflects the colder gas temperature within the compartment and hence lower overall pressure. The outlet gas velocity mainly depends on the pressure/temperatures within the compartment in under ventilated compartment fires and that explains the lower velocities observed at the top flow probes.

**Figure 8:**
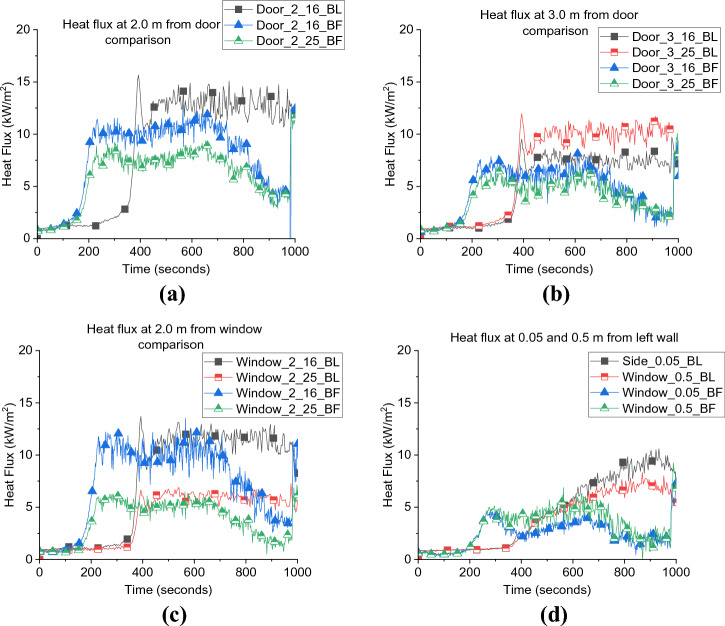
External radiative heat flux measurements (a) door at 2.0 m, (b) door at 3.0 m, (c) window and (d) side heat fluxes for both case_1 and BL

As presented in Figure 8a, b, the external heat fluxes from the door of BL case at 2.0 and 3.0 m were approximately 30 to 60% higher than BF. While the differences were around 20% opposite the window at both positions. As mentioned earlier, the higher gas temperatures within the compartment led to higher wall temperatures and radiation from the walls as shown in Figure 8d, the radiative heat flux from the side wall was approximately 100% higher from the BL scenario compared to the BF scenario.

The external heat flux reflects the effect of the internal fire dynamics differences between the two cases. The BL case experienced more efficient fresh air entrainment within the wood crib which led to a higher combustion efficiency and combustion products with higher temperatures. The hotter the fire, the higher the pressure and hence the external plume’s velocities were higher. This then appeared as higher radiative heat fluxes captured in front of the door, window and the side walls.

#### Numerical

In this section, the effect of the fuel location was further investigated using two FDS models constructed using the same methodology presented by Beshir et al. [15], to look at the BL and BF compartments before and at flashover. The FDS (FDS version 6.7.6) validated model for BL was presented in an earlier study [15], while the BF validation followed the same methodology and inputs [15] and its validation results are presented in this paper. The domain of each model was of 5.0 m × 6.0 m × 4.0 m (X, Y, Z) accompanied with a cell size of 0.06 m for the whole domain. The Heat Release Rate Per Unit Area (HRRPUA) of the wood (Pine wood) and cardboard were taken from the previous cone calorimeter study [7] under the heat flux of 75 kW/m^2^ for both materials. Similar to the values used in [15], the ignition temperature of the wood and cardboard, was set to 250°C and 260°C, respectively, the heat of combustion was taken as 20 MJ/kg for the dominating fuel (wood) and the soot yield was taken as 0.015. The accelerant/igniters (gasoline)’s HRRPUA curve used in the tests, was based on the early stage HRR curve of the experiments.

**Figure 9:**
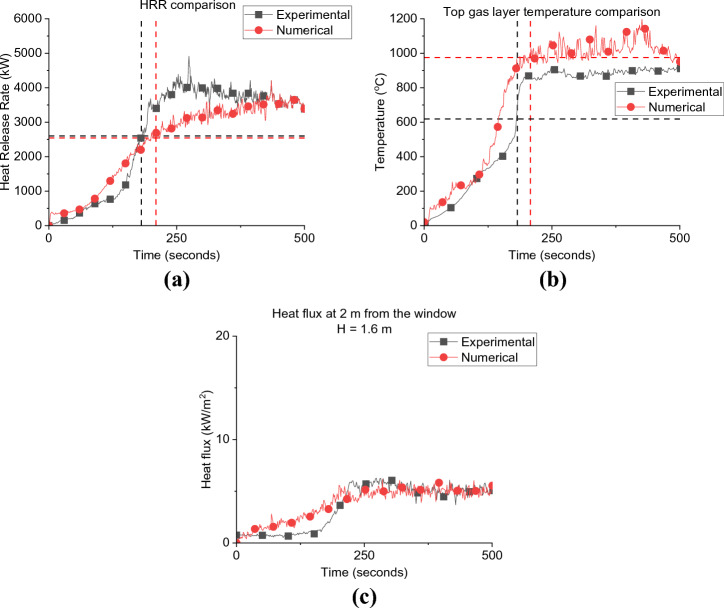
BF case validation: (a) Heat Release Rate, (b) Gas layer temperature, and (c) Window heat flux at 2.0 m to 1.6 m height (flashover for each case is marked with dashed lines)

**Figure 10:**
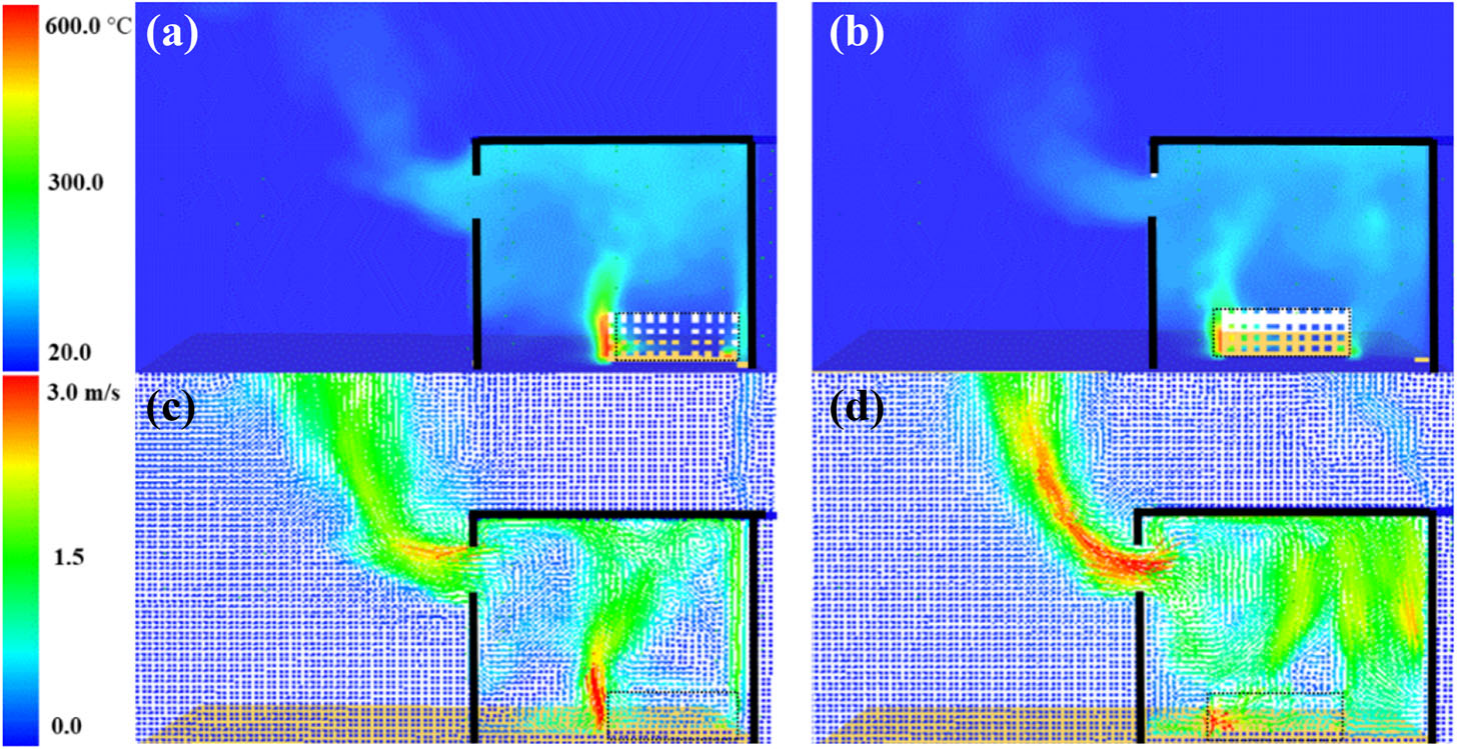
Averaged slice files over the first 50 s from ignition for: (a) BF case temperature, (b) BL case temperature, (c) BF case velocity, and (d) BL case velocity

The numerical flashover criteria is assumed to be the time when the heat flux measurements on the four corners of the compartment’s floor reaches 20 kW/m^2^ [24]. The focus was given to the pre-flashover period and this period was validated for the BF as presented in Figure 9. The time and heat release rate at flashover were found to be around 210 s/2250 kW, compared to experimental results of 180 s/2600 kW. The HRR-time curve was well replicated by the model before flashover and the averaged gas layer temperature at flashover was overestimated by around 15%. The heat flux from the window, at and around flashover, was also captured with relatively good accuracy. The BF model, along with the BL model, were used to further understand the observations from the experimental results. Figure 10 presents a temperature and velocity slice taken vertically in the middle of the compartment and averaged over the first 50 s from ignition. Figure 10a, b show that, compared to the BL case, the BF compartment experienced hotter gas layer temperatures, resulting from a relatively longer plumes at the front and back of the wood crib.

This is due to the poor air mixing within and around the wood crib in the BF case, as shown in Figure 10c, d. The BF case showed relatively high velocities only at the front and back of the wood crib, however, it shows very poor mixing (i.e., low gas velocities) within most part away from the crib. The BL scenario on the other hand showed very well distributed velocities within the compartment and around the crib. The poor mixing and air entrainment within the BF case led to quick spread over the front of the crib with a longer flame length compared to the BL case.

**Figure 11:**
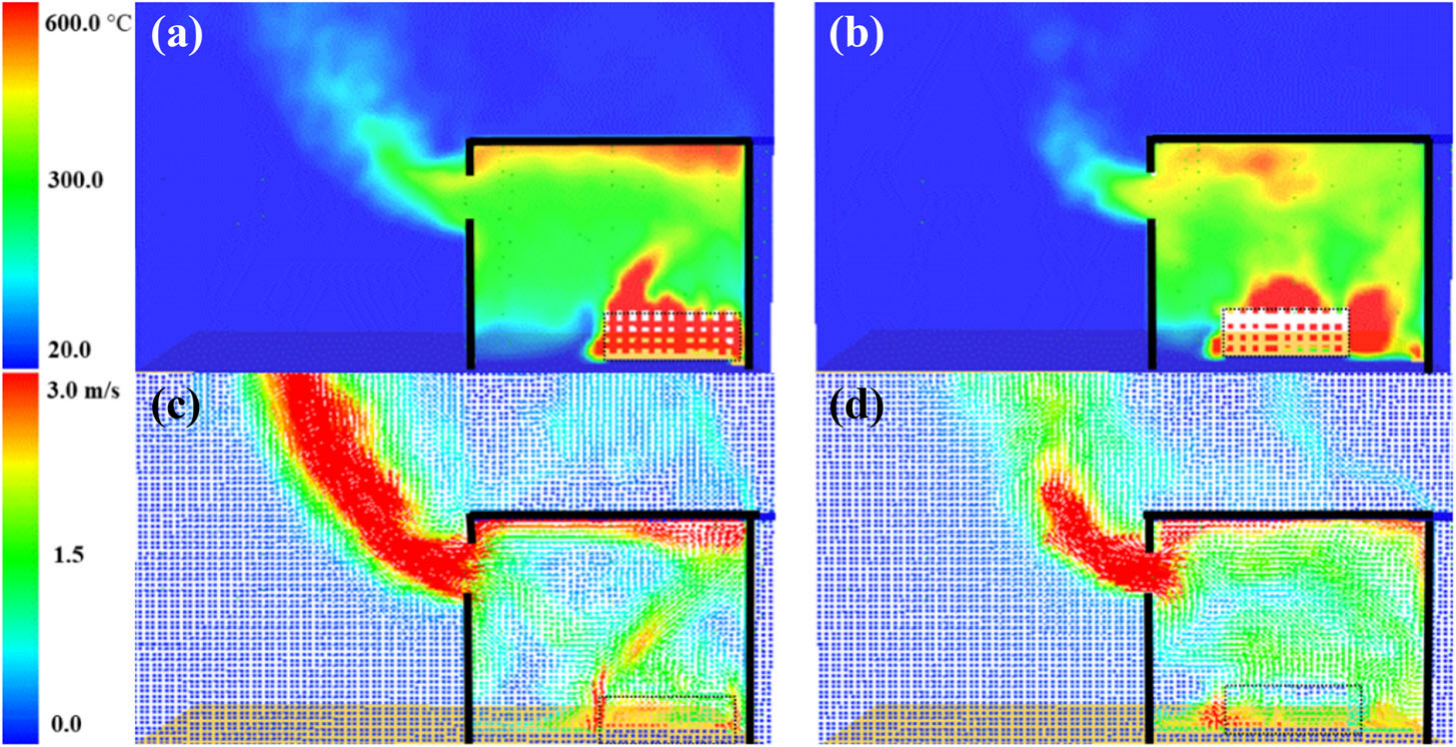
Averaged slice files over 150 s from ignition: (a) BF case temperature, (b) BL case temperature, (c) BF case velocity, and (d) BL case velocity

**Figure 12:**
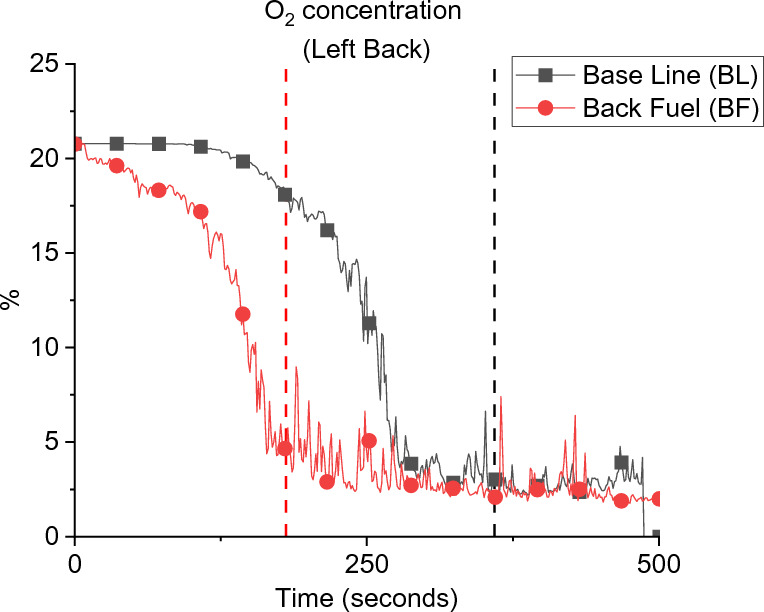
Comparison of the numerical oxygen concentration at the back of the BL and BF cases (flashover for each case is marked with dashed lines)

The cumulative effect of the air entrainment and mixing appeared clearly within both compartments after 150 s as presented in Figure 11. Where the BF compartment accumulated hotter and thicker gas layer and hence hotter walls as showed earlier in Figure 7. This effect led the BF compartment to reach flashover earlier than BL at around 200 s compared to 351 s and explains the experimental observations. In addition, Figure 12 demonstrates the numerical oxygen concentration within both compartments at the top left back point (same location as the LB TC10). As shown, the oxygen concentration within the top layer of the compartment dropped much earlier in the BF case due to the unburnt gases from the crib (due to the poor entrainment and mixing). The BL case, however, experienced almost ambient oxygen concentrations for more than 200 s, which shows that the burning of the wood crib was slower and of high efficiency due to the rich entrainment. Generally, placing the fuel package in the middle, compared to the back of the compartment, led to slower growth phase (e.g., flashover time was delayed by around 80%), hotter gas phase within the compartment, higher $\dot{q}_{fo}$, higher internal pressure, faster external plume velocity, and higher heat fluxes to the surroundings at different locations (e.g., 30 to 100% more). These results followed some of the conclusions by Hwang et al. [26] (e.g., for the time to flashover and the gas phase temperature), albeit, where Hwang et al. conducted compartment fires within a thermally thick bounded ISO-9705 room with heptane burner as fuel, compared to these thermally thin bounded compartments with cellulosic fuel.

### Ventilation Factor

In this section, the effect of the ventilation factor of two identical under-ventilated compartment fire experiments is investigated. The two cases are BF (window + door) and NW (door only), where the ventilation factors are 2.58 m^5/2^ and 2.26 m^5/2^, respectively.

**Figure 13:**
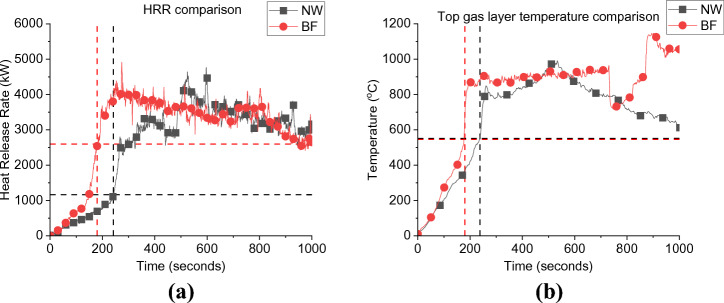
Fire dynamics (a) Heat Release Rate vs. time and (b) top gas layer temperature for both cases 1 and 2 (please note that for the BF case one of the walls collapsed around the 800 to 900 s and impacted the measured temperature in (b)

As presented in Figure 13, the time to reach flashover for the NW case was found to be at 248 s, which is around 70 s slower than the BF case (≈ 36%). Additionally, $\dot{q}_{fo}$ for the NW case (1165 kW) was found to be around 55% lower than that of the BF case, while the ventilation factor of the NW case is only around 12% less. This highlights the effect of the ventilation factor on the growth phase. This observation can be explained to be mainly due to the heat losses from the extra ventilation, therefore, the more the heat losses, the more energy is needed to heat up the compartment (top gas layer and walls) to reach flashover conditions. However, due to the restricted ventilation, the entrainment (air flow) around the wood crib in the NW scenario was not as consistent as in the BF scenario (with window and door). Therefore, even with less ventilation (i.e., less heat losses), it took more time for NW case to burn enough fuel and build up the required heat to reach flashover, mainly due to the air entrainment and distribution around the wood crib. The gas layer temperature was almost identical at the time to flashover, where the compartment was still fuel controlled. Post-flashover, once the compartment became ventilation-controlled, the BF scenario experienced slightly higher gas layer temperatures due to the better ventilation and hence higher combustion efficiency within the compartment. The maximum gas layer temperatures recorded were 900 and 845°C for the BF and NW cases, respectively. This was also reflected on the average HRR values at the steady post-flashover period, where the BF and NW cases averaged a HRR of approximately 4000 and 3200 kW, with the maximum values being approximately 4800 and 4500 kW, respectively.

The experimental results were also compared to the theoretical values from empirical equations (i.e., Eqs. 1, 2 and 3) used to estimate the maximum HRR (Max HRR) [24] and the maximum gas layer temperature ($T_{max}$) [27] within a given compartment.

1 $$Max HRR = 0.09 V_{f} \Delta H_{C}$$

2 $$T_{max} = \frac{{6000(1 - e^{ - 0.1\varphi )} }}{\sqrt \varphi }$$

3 $$\varphi = \frac{{A_{T} - A_{o} }}{{V_{f} }}$$

where $V_{f}$ is the ventilation factor; $\Delta H_{C}$ is the gross heat of combustion of wood (i.e., 17.5 MJ/kg) [28]; $A_{T}$ is the total area of the compartment internal surfaces; $A_{o}$ is the openings area. Theoretically, the maximum HRR within the BF and NW is estimated to be 4100 and 3600 kW compared to 4000 and 3200 kW found experimentally, respectively. Furthermore, the maximum gas layer temperature estimated theoretically within these compartments are 1210 and 1189°C compared to 930 and 972°C for the BF and NW, respectively.

**Figure 14:**
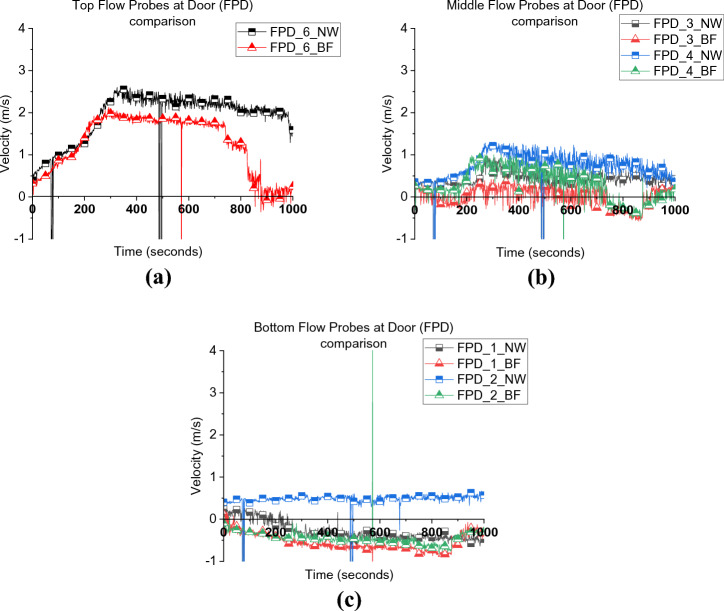
Door flow probes (a) top flow probe 6, (b) middle flow probes 3, 4 and (c) bottom flow probes 1, 2 for both BF and NW [Please note that FP_5 for NW was corrupted during the experiment and therefore not presented here]

**Figure 15:**
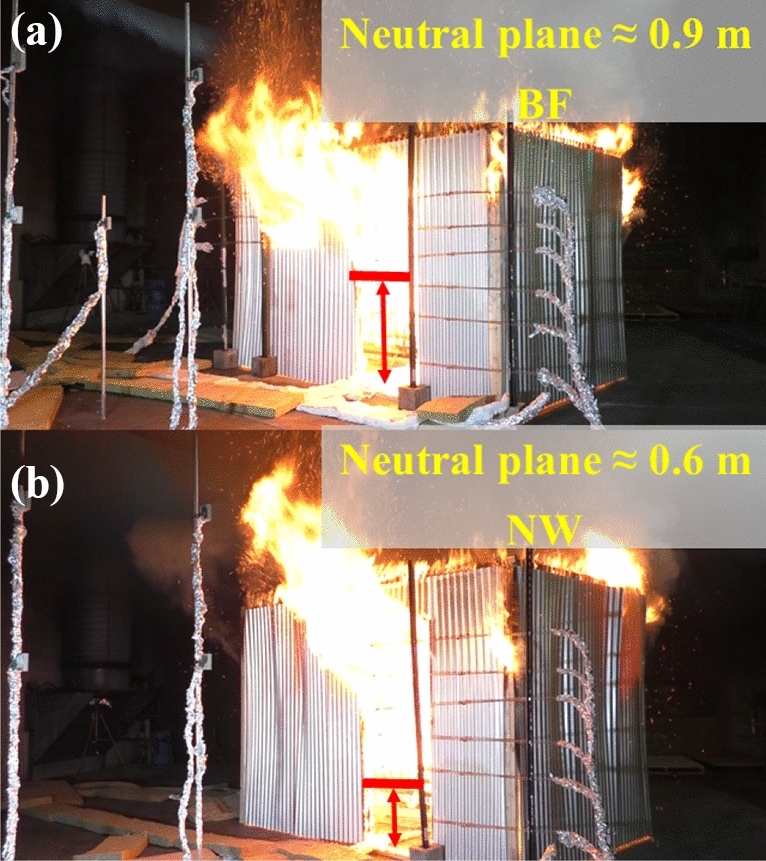
Post-flashover external plumes from (a) BF and (b) NW

As depicted in Figure 14, during the post-flashover period, due to the absence of the window for NW, the door was the only large vent present for the unburnt gases to leave the compartment. Therefore, the neutral plane (NP) for the NW scenario was around 0.6 m, which is around 30% less in height compared to the BF scenario which had a NP height of around 0.9 m, measured from the floor. This is also demonstrated in Figure 15 which shows the post-flashover period for both experiments.

**Figure 16:**
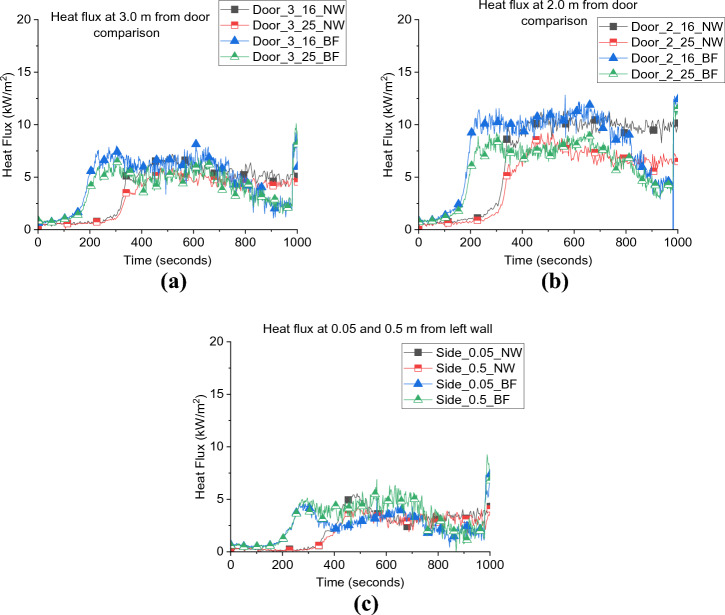
External radiative heat fluxes from the (a) door at 2.0 m, (b) door at 3.0 m and (c) side wall for both BF and NW

It was also found that the difference in the NP height and slight difference in the internal gas layer temperature did not affect the external heat flux from the door and side walls, as depicted in Figure 16. Generally, it was found that decreasing the ventilation factor by around 12.5%, the total heat losses were less and therefore, $\dot{q}_{fo}$ decreased by around 55% and the neutral plane was lower by around 30%, however, the external radiation was not significantly affected.

## Conclusions and Future Work

Three experiments were compared to study the effect of the fuel location and ventilation factor on the fire dynamics and external heat fluxes of under ventilated, thermally thin bounded compartment fires, with wood crib as a fuel load. The compartments were full-scale ISO-9705 with 0. 51 mm steel sheets walls. Detailed comparisons for the HRR, gas layer temperature, opening flow field and external heat fluxes were presented.

From the results, it was observed that locating the fuel at the back of the compartment caused a significant reduction in the time to flashover (defined in this work as external flaming) in comparison to the case with the fuel placed at the centre of the compartment. This is believed to be due to the restricted air entrainment experienced by the wood crib when attached to the wall. The fuel location, however, did not play an important role in changing the peak HRR and peak gas layer temperature. The velocity flow field from the openings was also found to be a bit higher for the outlet flow for the scenario where the wood crib was placed in the middle, which is expected due to the higher combustion efficiency that leads to higher average temperatures and pressure within the compartment. However, the estimated neutral plane for both cases was close in height. The experimental observations related to the fuel location effect were re-visited numerically. It was shown that, the fuel location can highly affect the air entrainment and mixing within the wood crib and the compartment, even at the fuel-controlled phase and thus affects the time/HRR at the flashover.

The ventilation factor was found to affect the HRR needed for flashover, the location of the neutral plane and the velocity of the top external gas flow (outlet flow). The higher ventilation factor caused higher HRR needed for flashover due to the higher heat losses through the extra ventilation. The time to flashover was slightly affected, however, the external radiative heat fluxes were almost similar in both cases.

Generally, placing the fuel package at the back of the compartment led to slightly cooler compartment fire, less time needed to reach flashover, higher $\dot{q}_{fo}$ and less radiative heat fluxes to the surroundings. Reducing the ventilation factor, mostly affected the fire pre-flashover and decreased the $\dot{q}_{fo}$, yet did not affect the external radiative heat flux.

The novelty of this work is twofold. Firstly, it focuses on investigating the influence of fuel location and ventilation on the severity of fires in informal settlement dwellings (ISDs). By specifically examining the time to reach flashover and the external heat flux from the dwelling of fire origin, this study addresses critical factors that contribute to fire spread in ISDs. This research adds to the existing body of knowledge by providing valuable insights into the fire dynamics within these complex structures. Secondly, the work introduces a methodology to replicate the experiments using computational fluid dynamics (CFD). By successfully replicating the experimental results, this study establishes a reliable tool for simulating fire scenarios in ISDs. The presented model enables further investigations into sensitivity analyses and real fire scenarios, generating important data for the development of fire spread models specific to informal settlements. The ability to accurately simulate and predict fire behaviour in ISDs is crucial for the development of effective fire mitigation strategies and the improvement of safety in these communities. Moreover, the subsequent study conducted by Lemmertz et al. [29], which builds upon the CFD models developed in this research, explores the effect of atmospheric wind on fire severity in ISDs, further demonstrating the versatility and applicability of CFD models in studying fire dynamics under various environmental conditions. As future steps, the refinement and expansion of the CFD models to encompass a broader range of scenarios and variables, such as different fuel types, ventilation conditions, and building materials, would contribute to advancing research on fire dynamics in ISDs, enhancing fire safety strategies, and improving the resilience of informal settlements to fire incidents. The work presented within this paper will also aid engineers and urban planners in understanding and potentially mitigating urban informal settlement fires by providing an understanding of the phenomena within these complex structures, thus allowing evidence-based decisions to occur.
